# Editorial: Biomaterial advances in cartilage and meniscus regeneration

**DOI:** 10.3389/fbioe.2022.1054268

**Published:** 2022-10-17

**Authors:** Liwei Fu, Weimin Guo, Quanyi Guo

**Affiliations:** ^1^ Beijing Key Laboratory of Regenerative Medicine in Orthopedics, Key Laboratory of Musculoskeletal Trauma & War Injuries PLA, Institute of Orthopedics, Chinese PLA General Hospital, Beijing, China; ^2^ School of Medicine, Nankai University, Tianjin, China; ^3^ Guangdong Provincial Key Laboratory of Orthopedics and Traumatology, Department of Orthopaedic Surgery, First Affiliated Hospital, Sun Yat-sen University, Guangzhou, China

**Keywords:** cartilage, meniscus, regeneration, biomaterial, osteoarthritis

Due to the absence of vascular, lymphatic, and neural tissues, articular cartilage and meniscus have limited ability to regenerate after a defect occurs. Without timely treatment and repair cartilage and meniscal deficiency progresses to osteoarthritis (OA), which places enormous burdens on patients and the social health system. With the rapid development of tissue engineering and regenerative medicine, biomaterials have been used to reconstruct cartilage and meniscus defects and for the anti-inflammatory treatment of early OA. Biomaterials are natural or man-made materials used to replace lost cartilage or meniscus structures with the goals of restoring morphology and function or providing anti-inflammatory treatment during cartilage and meniscus regeneration. The diversity of biomaterials and the flexibility of combinations have shown great potential for the treatment and regeneration of cartilage and meniscus.

Given the indispensable role of biomaterials in cartilage and meniscus regeneration, we prepared this Research Topic to summarize the progress in this field. Three review articles in this Research Topic summarize the application and development of different types of advanced biomaterials. Biocompatibility has always been a necessary characteristic of biomaterials. Decellularized cellular-extracellular matrix (ECM) not only retains the components of cartilage tissue to the greatest extent but also stores many bioactive factors; thus, ECM has a natural advantage in articular cartilage regeneration. Zhang et al. have demonstrated the promising future of ECM in articular cartilage regeneration. They also proposed ECM-based biological scaffolds that adapt to the environment over time as a future research hotspot.

Nanomaterials, due to their bioactive properties and programmable surface properties, can be designed to produce a variety of personalized implants with increased accuracy. Chen have introduced nanotechnology-based implants for bone and cartilage repair. They believe that although nanotechnology has great innovative uses in the field of orthopedic implants, further biosafety studies are necessary. Furthermore, as biomaterials with unique 3D mesh structures, hydrogels have been widely used in the treatment of OA due to their good encapsulation, biodegradability, and favorable microenvironment for cell growth. Wang et al. recently reviewed the application of hydrogel-based scaffolds for the treatment of osteoarthritis. The literature has also proposed different hydrogel design strategies based on osteoarthritis classification as early or advanced stages. Extracellular vesicles, as a kind of lipid bilayer structure, is a promising stem cell replacement strategy because of their biological activity and functionality. Lin et al. described the global research trends in extracellular vesicles based on stem cells from 1991 to 2021 through a bibliometric and visualized study. Finally, we describe an interesting study on the small molecule compound XMU-MP-1 in the treatment of osteoarthritis. Hao et al. reported that, as a selective MST1/2 inhibitor, XMU-MP-1 showed a protective effect on chondrocytes and the extracellular matrix in an OA model; thus, this compound may be a promising treatment for OA.

In conclusion, we believe that the studies and reviews in this Research Topic provide new insight into the choice of biomaterials for engineering solutions for articular cartilage and meniscus regeneration, which helps us to better understand the concept of regeneration.

